# 

**Published:** 1847-08

**Authors:** 


### CONTENTS

OF NO. II.

---

### ORIGINAL COMMUNICATIONS.

ESSAYS AND CASES.

ART. I.—Observations on Cancrum Oris, as it appeared in the Cincinnati Orphan Asylum, from February, 1842, to May, 1847. By THOMAS CARROLL, M.D., of Cincinnati, Ohio. - - - - - - - - 93

ART. II.—Notes on Medical Matters and Medical Men in Paris. By DAVID W. YANDELL, M. D., of Louisville, Ky. - - - - - - - - - - 128

REVIEWS.

ART. III.—Expériences Relative aux Effets de l'Inhalation de l'Ether Sulphurique sur la Système Nerveux. Par F. A. LONGET, Professeur d'Anatomie et de Physiologie, etc., etc. Mémoire lu à l'Académie Royale de Médecine. Victor Massou, Paris 1847. pp. 54. 8vo.

Experiments relative to the Effects of the Inhalation of Sulpuric Ether upon the Nervous System. Read to the Royal Academy of Medicine. By F. A. LONGET. - 153

SELECTIONS FROM AMERICAN AND FOREIGN JOURNALS.

Code of Medical Ethics. - - - - - - - - - 165

### ORIGINAL INTELLIGENCE.

Health of the City. - - - - - - - - - - 179
Improved Water-Pump. - - - - - - - - 180
Geological Survey. - - - - - - - - - - 180
Adulterations in Medicine. - - - - - - - 181
Murphy's Parturition. - - - - - - - - 181
Length of Lecture Terms. - - - - - - - 182
Proceedings of the National Medical Convention. - - 183
Medical Department of the University of Louisville. - 184
Postage on this Journal. - - - - - - - - 184

